# Prevalence of Nasal *Staphylococcus aureus* Carriage in Patients Undergoing Hemodialysis and Assessment of Risk Factors: A Cross-Sectional Study of Outpatients at a University Hospital

**DOI:** 10.3390/healthcare13030245

**Published:** 2025-01-26

**Authors:** Daniella Teixeira Bezerra, Raquel Agnelli Mesquita-Ferrari, Kristianne Porta Santos Fernandes, Sandra Kalil Bussadori, Lara Jansiski Motta, Ellen Sayuri Ando-Suguimoto, Céline Frochot, Alessandra Messina Perini, Flavia Rossi, Marcelo Jenne Mimica, Benedito Jorge Pereira, Anna Carolina Ratto Tempestini Horliana

**Affiliations:** 1 Postgraduate Program in Biophotonics-Medicine, University Nove de Julho, UNINOVE, São Paulo 01504-001, Brazil; daniellateb@hotmail.com (D.T.B.); raquel.mesquita@gmail.com (R.A.M.-F.); kristianneporta@gmail.com (K.P.S.F.); sandrakalil@uni9.pro.br (S.K.B.); larajmotta@uni9.pro.br (L.J.M.); esa.2406@gmail.com (E.S.A.-S.); 2 Postgraduate Program in Rehabilitation Sciences, University Nove de Julho, UNINOVE, São Paulo 01504-001, Brazil; 3 Université de Lorraine, CNRS, LRGP, F-54000 Nancy, France; celine.frochot@univ-lorraine.fr; 4 Clinical Microbiology Laboratory, Clinical Hospital, University of Sao Paulo Medical School, São Paulo 01246-000, Brazil; alessandraperini@hotmail.com (A.M.P.); flaviarossi61@gmail.com (F.R.); 5 Division of Microbiology, Department of Pathological Sciences, Santa Casa de São Paulo School of Medicine, São Paulo 01224-001, Brazil; mjmimica@hotmail.com; 6 Renal Division, Internal Medicine, Clinical Hospital, University of Sao Paulo Medical School, São Paulo 05403-010, Brazil; beneditojp@gmail.com

**Keywords:** *Staphylococcus aureus*, chronic kidney disease, oxacillin-resistant strains

## Abstract

**Background/Objectives:** Infection poses a significant threat of mortality in patients with chronic kidney disease (CKD) undergoing hemodialysis. *Staphylococcus aureus* (*S. aureus*) is a common etiological agent, with prior nasal colonization identified as a risk factor for infection. The aim of the present cross-sectional study was to determine the prevalence of nasal *S. aureus* carriage among patients with CKD undergoing dialysis at a university hospital and identify potential factors associated with colonization. **Methods:** Nasal swabs were obtained, and bacterial isolates were identified using matrix-assisted laser desorption/ionization time-of-flight spectrometry and antibiogram testing with the Vitek 2 system. Demographic and clinical data were collected for the investigation of risk factors associated with colonization. **Results:** Among the 96 patients analyzed, 34 were carriers of *S. aureus.* Among these carriers, three (8.8%) harbored oxacillin-resistant strains. More than half of the *S. aureus* strains exhibited resistance to clindamycin but susceptibility to oxacillin. Colonization was associated with age and the use of corticosteroids/immunosuppressants. **Conclusions:** The prevalence of nasal *S. aureus* carriage was high among patients undergoing dialysis at the university hospital, exceeding that found in the general population. Nasal colonization by *S. aureus* was linked to corticosteroid use and age. Understanding factors associated with *S. aureus* nasal colonization in patients on dialysis can assist healthcare providers in preventing the spread of infection and cross-contamination, while reducing risk in this population.

## 1. Introduction

Patients undergoing hemodialysis are predisposed to healthcare-associated infections, which are associated with high morbidity and mortality rates [1,2,3]. This susceptibility arises from the dialysis procedure itself as well as the deficient innate and adaptive immunity found in this population [4,5,6]. Moreover, bacterial virulence and adherence properties are crucial to the pathogenesis of infection in these patients [7]. *Staphylococcus aureus* (*S. aureus*) is the most prevalent etiological agent in vascular access-related infections among patients on dialysis, with a mortality rate of around 25% [8,9,10].

Nasal colonization by *S. aureus* is a recognized risk factor for the development of infections caused by this microorganism [11,12,13,14,15] and is also a potential source of bacterial dissemination in the environment, promoting person-to-person transmission through direct or indirect contact [11,12,15]. The risk of bloodstream infection among patients with CKD undergoing dialysis who are nasal carriers of methicillin-sensitive *S. aureus* (MSSA) is four times higher compared to non-carriers [8,16]. Phage typing, which is a laboratory method used to characterize the susceptibility of different strains of microorganisms to specific bacteriophages, and plasmid DNA analysis have demonstrated that 80–90% of *S. aureus* infections in such patients originate endogenously, meaning that the causative agent of infection is already colonizing the individual [8,11,12,15]. Although we are exposed to *S. aureus* on a daily basis, only a few individuals are long-term carriers [17]. Longitudinal studies distinguish distinct patterns of nasal *S. aureus* carriage in healthy individuals: persistent (10–35%), intermittent (20–75%), and non-carrier (5–50%) [6,17,18,19].

The prevalence and incidence of nasal *S. aureus* carriage vary depending on the population studied [20]. Patients with CKD undergoing hemodialysis often have multiple risk factors for bacterial colonization, such as diabetes, advanced age, repeated exposure to healthcare procedures, prolonged hemodialysis, use of dialysis catheters, and frequent exposure to antibiotics [14,21,22]. Consequently, the prevalence of nasal *S. aureus* carriage in such individuals is high, with rates ranging from 27 to 60% in different studies [11,12,14,22].

Nasal colonization by *S. aureus* is highly prevalent among patients undergoing dialysis and poses a significant risk of infection, particularly bacteremia. Such infections are associated with high mortality and morbidity rates. However, there is a lack of studies exploring potential factors linked to this colonization. Therefore, further research on such factors is essential and will assist in identifying patients with a greater likelihood of nasal colonization by this pathogen. The present study is a cross-sectional subset of a pilot study comparing treatments for nasal decolonization among patients undergoing dialysis who are carriers of nasal *S. aureus* [23]. The aim was to determine the prevalence of nasal *S. aureus* carriage in patients with CKD at a hemodialysis unit of a university hospital and identify potential factors associated with colonization.

## 2. Materials and Methods

### 2.1. Materials

- Nasal Swabs: Sterile swab-type collectors (Absorve^®^, Cralplast, Cotia, SP, Brazil) for nasal sample collection.
- Transport Medium: Tubes containing Stuart medium for transporting swab samples to the laboratory.
- Culture Media: Blood agar plates.
- Mass Spectrometry Matrix: α-Cyano-4-hydroxycinnamic acid (HCCA) for ionization in MALDI-TOF analysis.
- Chemical Reagents for Matrix Preparation: This study used a preprepared matrix. No acid was included in the matrix composition.
- Mass Spectrometry Equipment: MALDI-TOF system (bioMérieux^®^, Marcy-l’Étoile, France) for bacterial identification.
- Antimicrobial Susceptibility Testing System: Vitek 2 system (bioMérieux^®^), including AST-P637 cards for Gram-positive cocci.
- Quality Control Strains: *Staphylococcus aureus* ATCC 29213, *Candida glabrata* ATCC MYA-2950, *Klebsiella aerogenes* ATCC 13048, and *Escherichia coli* ATCC 25922 to validate test accuracy.
- Antibiotics Tested: Tigecycline, clindamycin, vancomycin, erythromycin, teicoplanin, gentamicin, levofloxacin, sulfamethoxazole–trimethoprim, linezolid, rifampicin, penicillin, and oxacillin.
- Densitometer: Used to standardize bacterial suspension turbidity at 0.5–0.63 McFarland units (DensiChek™, bioMérieux^®^, Marcy-l’Étoile, France) [24,25,26].

### 2.2. Study Duration and Population

This cross-sectional study received approval from the Institutional Review Board of Nove de Julho University (certificate number: 3505587) and the Institutional Review Board of the hospital affiliated with the School of Medicine of the University of São Paulo (certificate number: 3645881). Individuals undergoing treatment were recruited from the hemodialysis service, and those who agreed to participate signed a statement of informed consent. The authors followed the STROBE checklist for cross-sectional studies. Surveys were conducted during hemodialysis sessions at the hospital from November 2019 to November 2021. Biological samples collected during these sessions were sent to the Central Laboratory Division—Microbiology Section at the hospital. The samples were processed using standard microbiological methods, including cultures. Colonies displaying typical characteristics of *S. aureus* were transferred to an additional plate for bacterial identification using MALDI-TOF (bioMérieux^®^).

### 2.3. Sample Size Calculation

A non-probabilistic convenience sample based on accessibility or availability was used to estimate the prevalence of nasal *S. aureus* carriage in patients with CKD receiving treatment at the hemodialysis service. In total, 113 patients were initially recruited, 17 of whom declined to participate in this study. Thus, 96 patients were screened for *S. aureus*.

### 2.4. Eligibility Criteria

Male and female patients with CKD 18 years of age or older undergoing hemodialysis at the university hospital were included. Patients with active *S. aureus* infection, those with no nasal disease or obstruction, and those involved in other studies were excluded.

### 2.5. Questionnaire Addressing Factors Associated with S. aureus Colonization

**Figure 1:**
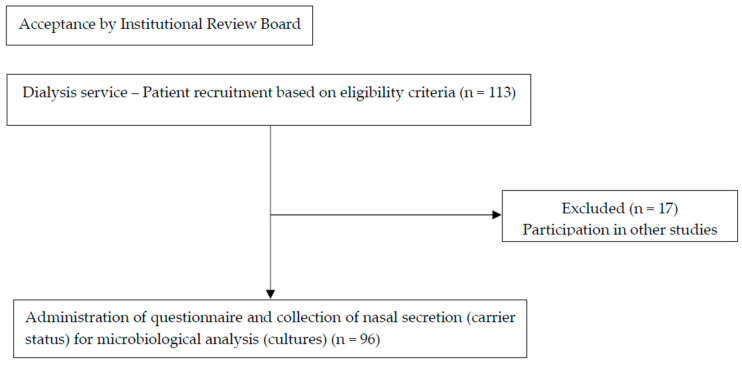
Flowchart illustrating recruitment and data collection process. Among 113 patients recruited based on eligibility criteria at dialysis service, 17 were excluded due to declining participation or involvement in other studies. Ninety-six patients completed the questionnaire and provided nasal secretion samples for microbiological analysis (cultures).

Clinical and demographic data were collected from all 96 participants to assess potential factors associated with nasal *S. aureus* carriage in this population. These factors included age, sex, marital status, occupation, education, housing, etiology of CKD, comorbidities, duration of dialysis treatment before initiation of the protocol, presence of smoking, type and duration of venous access, history of kidney transplantation, systemic antibiotic therapy in the previous 12 months, hospitalization, and infection (including skin infection) in the previous 12 months. The steps of Section 2.2, Section 2.3, Section 2.4, Section 2.5 and Section 2.6 are schematically summarized in Figure 1.

### 2.6. Isolation of S. aureus in Recruited Patients

**Figure 2:**
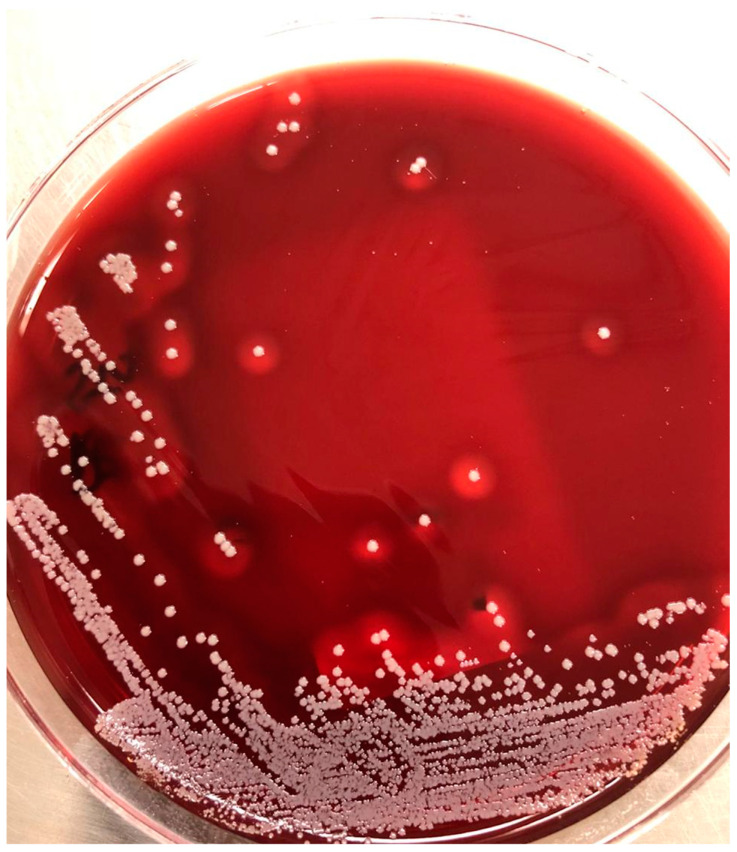
Streaking on blood agar plate.

For the detection of nasal *S. aureus*, trained researchers collected samples from patients who met the eligibility criteria using sterile swab-type collectors (Absorve^®^). For the procedures, two trained researchers equipped with nasal swabs collected samples in different shifts (morning and afternoon) and on different days (Monday to Saturday) of hemodialysis treatment. A standardized technique was employed: five gentle circular movements in each anterior nostril [22]. The same swab was used in both nostrils (Figure 2). The swabs were transported in tubes containing Stuart medium within four hours and sent to the microbiology laboratory.

All swabs were streaked on blood agar plates. Colonies typical of *S. aureus* were transferred to another plate for bacterial identification using MALDI-TOF (bioMérieux^®^). In simple terms, these colonies were placed on a polymeric matrix plate, followed by laser desorption–ionization mass spectrometry to identify bacteria.

The procedure was as follows: Suspected colonies of *S. aureus* grown on a blood agar plate are transferred to a target slide in small spots. A drop of α-cyano-4-hydroxycinnamic acid (CHCA) matrix, supplied by bioMérieux, is applied to each spot and left to dry at room temperature. Once the matrix crystallizes, the slide is placed in the ionization chamber of a mass spectrometer. Ultraviolet laser pulses desorb and ionize the sample molecules, which are then accelerated through an electrostatic field and into a vacuum tube. Smaller ions reach the detector faster than larger ones, and their time-of-flight (TOF) is used to calculate *m*/*z* values. The resulting mass spectrum is known as a proteomic fingerprint and is compared to a database of spectra from well-characterized microorganisms for identification.

The analysis was performed using the Vitek MS system, with data processing handled by the Myla software program (bioMérieux^®^, Marcy-l’Étoile, France, version [4.8]), both from bioMérieux. Each spot analysis took approximately one minute. Slides contained three groups of 16 spots each. Quality control was ensured by including *Candida glabrata* ATCC MYA-2950, *Klebsiella aerogenes* ATCC 13048, and a negative control on every slide. Additionally, *Escherichia coli* ATCC 25922 was used as a mandatory control strain for reading the slide.

Antimicrobial susceptibility testing was conducted using the Vitek 2 system (bioMérieux^®^). The antimicrobials investigated were tigecycline, clindamycin, vancomycin, erythromycin, teicoplanin, gentamicin, levofloxacin, sulfamethoxazole–trimethoprim, linezolid, rifampicin, penicillin, and oxacillin. The results were reported following the guidelines of the Clinical and Laboratory Standards Institute (CLSI) (M100-Ed 29/30) [11].

Antimicrobial susceptibility testing (AST) was carried out using the Vitek System; isolated colonies from the same blood agar plate used for the MALDI-TOF identification were also utilized for the AST. For Gram-positive cocci, the AST-P637 card was employed. A bacterial suspension was prepared in sterile saline and adjusted to a specific density (0.50–0.63 McFarland units) using the DensiCheck system (bioMérieux). The AST card contained predefined concentrations of antimicrobial agents in microdilution wells and was inoculated with the bacterial suspension via a cannula under vacuum in the equipment and then incubated at a controlled temperature (35–37 °C) for up to 18 h.

The Vitek System (bioMérieux^®^, Marcy-l’Étoile, France, version [4.8]) monitors bacterial growth at each antimicrobial concentration using optical density measurements to determine the minimum inhibitory concentration (MIC) for each drug. The results are interpreted by the accompanying software program based on the guidelines of the Clinical and Laboratory Standards Institute (CLSI), classifying each antimicrobial as susceptible, intermediate, or resistant. Quality control is ensured using strains (e.g., Staphylococcus *aureus* ATCC 29213). The AST-P637 card tests various antibiotics, such as penicillin, oxacillin, cephalosporins, vancomycin, teicoplanin, levofloxacin, erythromycin, clindamycin, tigecycline, and linezolid.

### 2.7. Statistical Analysis

The Kolmogorov–Smirnov test was used to assess normality. Data were expressed as mean and standard deviation (SD) or median and interquartile range (IQR). Either Student’s *t*-test or the Mann–Whitney test was used to compare quantitative variables. Nominal qualitative variables were compared using either the chi-square test or Fisher’s exact test. A logistic regression model was adjusted to determine possible associations between nasal colonization by *S. aureus* and age, sex, education level, comorbidities, use of catheter/fistula, antibiotic use, duration of dialysis therapy, previous hospitalization, and infection (*p* < 0.10). All analyses were conducted using SPSS v. 18 (IBM Corp., Armonk, NY, USA). A *p*-value < 0.05 was considered indicative of statistical significance.

## 3. Results

In total, 96 patients with CKD undergoing hemodialysis were included in the present study; 35 (35%) were determined to be nasal carriers of *S. aureus* (Table 1).

The mean age was 45 years (standard deviation ± 17.47). Men comprised 49% of the participants. The predominant duration of dialysis was one to five years. Factors associated with colonization were younger age and corticosteroid/immunosuppressant therapies (Table 1). The distribution of other demographic characteristics, comorbidities, and clinical characteristics was similar between non-carriers and carriers. A total of 3 out of the 34 (8.8%) nasal *S. aureus* carriers were colonized with oxacillin-resistant strains (MRSA). Table 2 displays the characteristics of the patients colonized with MRSA.

Among the 34 nasal *S. aureus* carriers, more than 50% of the strains of *S. aureus* were resistant to clindamycin but susceptible to oxacillin (MSSA). The results of the antimicrobial susceptibility testing of *S. aureus* strains (n = 34) indicated that 55.8% of the strains were resistant to clindamycin, while 44.2% were susceptible; 64.7% of the strains were resistant to erythromycin, while 35.3% were susceptible. None of the strains were resistant to gentamicin, levofloxacin, linezolid, rifampicin, teicoplanin, tigecycline, or vancomycin. Resistance to oxacillin was found in 8.83% of the strains, with 91.17% susceptible. All strains (100%) were resistant to penicillin. Resistance to trimethoprim–sulfamethoxazole (TMP-SMX) was 2.9%, with 97.1% being susceptible. No strains had intermediate resistance profiles for any of the antimicrobials tested (Table 3).

## 4. Discussion

Our results revealed that 3 of the 34 nasal carriers of *S. aureus* (8.8%) were colonized with oxacillin-resistant strains (MRSA). Additionally, more than half of the *S. aureus* strains were resistant to clindamycin while being susceptible to oxacillin (MSSA). Regarding risk factors, age and the use of corticosteroids and immunosuppressants were associated with colonization.

Evidence indicating nasal colonization by *S. aureus* as an independent risk factor for infection by this agent in the population with CKD undergoing hemodialysis is extensively documented in the literature [8,9,10,16,27,28,29]. However, few studies have investigated the risk factors associated with nasal colonization in carriers of *S. aureus* undergoing hemodialysis for CKD [12].

Over the years, the prevalence of nasal *S. aureus* carriage has changed, which is attributed to improvements in personal hygiene, shifts in socioeconomic status, and smaller family sizes [12].

We investigated the prevalence of nasal *S. aureus* carriage in patients with CKD undergoing hemodialysis and identified a rate of 35%. The literature reports *Staphylococcus aureus* carriage rates ranging from 15% to 55% in healthy adult populations [30,31,32]. This divergence may reflect differences in study populations, screening methods, and microbiological analysis.

In the present study, the prevalence of MRSA strains was 8.8% among patients with CKD undergoing hemodialysis. The rates identified in two meta-analyses were 7.1% [33] and 12.8% [21]. These two studies found higher MRSA colonization rates in patients with CKD undergoing hemodialysis compared to the prevalence of MRSA colonization in the general population reported in a study conducted by the US Centers for Disease Control and Prevention between 2001 and 2004, which ranged from 0.8 to 1.5% [34].

Regarding patients with MRSA colonization (3/34), two shared a potential associated factor: prolonged hemodialysis duration (>120 months). Prolonged treatment may increase patient exposure to *S. aureus*, contributing to higher colonization rates. The findings of a previous study [34] are consistent with our results. However, additional evidence is required to confirm this association. The third MRSA carrier was a young adult (19 years of age) on dialysis for less time (30 months). However, this patient had a history of previous *S. aureus* infections in the tunneled hemodialysis access site and had used antibiotics in the previous 12 months to treat the infections. In previous studies, these two factors were reported to be associated with MRSA colonization in patients with CKD undergoing hemodialysis [35,36]. The three MRSA results were promptly conveyed to the infection prevention and control unit of the hospital for appropriate action. The medical team also monitored and managed the affected patients to ensure proper care and adherence to infection control protocols.

The high prevalence of methicillin-resistant *S. aureus* (MRSA) in patients with chronic kidney disease (CKD) undergoing hemodialysis probably results from frequent healthcare exposure, the use of vascular access devices, and recurrent hospitalizations. Prolonged antibiotic treatments and comorbidities, such as diabetes and hypertension, further increase susceptibility to MRSA colonization and infection. These factors underscore the importance of robust infection control measures and antibiotic stewardship programs [37].

Of note, 55.8% of the *S. aureus* isolates had a high rate of resistance to clindamycin, which was much higher than that found for oxacillin (8.8%). The clindamycin-resistant isolates were all susceptible to oxacillin (MSSA). Studies by Vicetti et al. [38] and Khamash et al. [39] also found high rates of clindamycin resistance in the *S. aureus* isolates. Clindamycin resistance results from selective pressure when this antibiotic is used frequently for the empirical management of *S. aureus* infection and from the dissemination of determinants of clindamycin resistance (erm genes) [35,36].

Regarding risk factors, we identified an association between younger age and nasal colonization of *S. aureus* in patients with CKD undergoing hemodialysis. A cross-sectional study conducted by Diawara et al. [40] reported a higher colonization rate among individuals aged 18 to 35 years (young adults), followed by those aged 36 to 50 years (adults) and 51 to 83 years (older adults). Conversely, Saxena et al. [41] found that nasal *S. aureus* carriage was higher among individuals aged 75 to 84 years, followed by those aged 65 to 74 years. This variation suggests that other factors, such as differences in immune conditions, exposure to community or hospital environments, or differences in study methods, may influence the association between age and *S. aureus* colonization.

We also identified an association between the nasal *S. aureus* carriage in patients with CKD and the chronic use of corticosteroids or immunosuppressants. This association may be explained by the exogenous use of glucocorticoids, which may elevate endogenous cortisol levels, suppressing the innate immune system and exerting an impact on adaptive immunity and inflammation [42]. One study demonstrated that polymorphisms in the glucocorticoid receptor gene are significantly linked to nasal *S. aureus* carriage [39].

Our study has several limitations that should be considered. Nasal *S. aureus* colonization was determined based on a single nasal secretion sample per participant. Thus, it was not possible to determine whether positive results corresponded to intermittent or persistent nasal carriage. The genetic lineage diversity of the strains was not investigated. Screening for *S. aureus* carrier status was limited to the nasal cavity without investigating other important sites, such as the throat. Additionally, the small sample size may have limited the detection of other factors associated with nasal colonization by *S. aureus* in this population.

## 5. Conclusions

As the present study was conducted at a single center with a specific population (patients with chronic kidney disease undergoing hemodialysis), the findings cannot be generalized to all patients with CKD on peritoneal dialysis, those in other stages of CKD, or to other healthcare facilities. In conclusion, the prevalence of nasal colonization by *S. aureus*, including MRSA strains, was high in this cross-sectional study involving dialysis patients. This colonization may be associated with age and corticosteroid use. Further multicenter studies with larger sample sizes are needed to investigate additional risk factors associated with nasal *S. aureus* carriage in the population undergoing dialysis.

## Figures and Tables

**Table 1:** Demographic and clinical characteristics of participants.

Variable	*Staphylococcus aureus* Carrier			*p*
	No	Yes	Total	
	62 (65%)	34 (35%)		
Age (years)	49.79 ± 17.11	39.06 ± 16.14	45.99 ± 17.47	0.003 *
Sex (male)	33 (53.2%)	14 (41.2%)	47 (49%)	0.259 **
Education				0.798 **
Primary school	13 (21.3%)	8 (23.5%)	21 (22.1%)	
High school	33 (54.1%)	16 (47.1%)	49 (51.6%)	
University	15 (24.6%)	10 (29.4%)	25 (26.3%)	
Number of residents in home				0.095 **
1	6 (9.7%)	4 (11.8%)	10 (10.4%)	
2	22 (35.5%)	5 (14.7%)	27 (28.1%)	
≥3	34 (54.8%)	25 (73.5%)	59 (61.5%)	
Number of rooms in home				0.893 ***
1–2	3 (4.8%)	1 (2.9%)	4 (4.2%)	
3–4	26 (41.9%)	15 (44.1%)	41 (42.7%)	
≥5	33 (53.2%)	18 (52.9%)	51 (53.1%)	
Employment status				0.221 ***
On sick leave	0 (0%)	1 (2.9%)	1 (1%)	
Retired	17 (27.4%)	6 (17.6%)	23 (24%)	
Unemployed	6 (9.7%)	4 (11.8%)	10 (10.4%)	
Homemaker	7 (11.3%)	3 (8.8%)	10 (10.4%)	
Student	3 (4.8%)	6 (17.6%)	9 (9.4%)	
Formal employment	29 (46.8%)	14 (41.2%)	43 (44.8%)	
Income (basic salary) ^#^				0.493 **
1 to 2	28 (59.6%)	15 (60.0%)	43 (59.7%)	
3 to 4	11 (23.4%)	8 (32.0%)	19 (26.4%)	
5 or more	8 (17.0%)	2 (8.0%)	10 (13.9%)	
Hypertension	33 (53.2%)	16 (47.1%)	50 (52.1%)	0.563 **
Stroke	3 (4.8%)	5 (14.7%)	8 (8.3%)	0.127 ****
Chronic liver disease	6 (9.7%)	2 (5.9%)	8 (8.3%)	0.708 ****
Dyslipidemia	4 (6.5%)	2 (5.9%)	6 (6.3%)	1.000 ****
Heart failure	3 (4.8%)	2 (5.9%)	5 (5.2%)	1.000 ****
Diabetes mellitus	18 (29%)	6 (18.2%)	24 (25.3%)	0.247 **
Previous *S. aureus* infection	4 (6.5%)	4 (11.8%)	8 (8.3%)	0.448 ****
Infection (previous 12 months)	37 (59.7%)	16 (47.1%)	53 (55.2%)	0.234 **
Antibiotic use (previous 12 months)	35 (56.5%)	14 (41.2%)	49 (51%)	0.152 **
Hospitalization (previous 12 months)	15 (24.2%)	5 (14.7%)	20 (20.8%)	0.274 **
Transplant	18 (29%)	9 (26.5%)	27 (28.1%)	0.789 **
Corticosteroid or immunosuppressor (previous 3 months)	4 (6.5%)	9 (26.5%)	13 (13.5%)	0.011 ****
Duration of dialysis				0.570 ***
<1 year	6 (9.7%)	6 (17.6%)	12 (12.5%)	
1–5 years	24 (38.7%)	12 (35.3%)	36 (37.5%)	
5–10 years	16 (25.8%)	9 (26.5%)	25 (26.0%)	
10–20 years	7 (11.3%)	5 (14.7%)	12 (12.5%)	
>20 years	9 (14.5%)	2 (5.9%)	11 (11.5%)	
Catheter/fistula				0.406 **
Arteriovenous fistula	40 (64.5%)	19 (55.9%)	59 (61.5%)	
Tunneled hemodialysis catheter	22 (35.5%)	15 (44.1%)	37 (38.5%)	

MMW = monthly minimum wage, * Student’s *t*-test, ** chi-square test, *** likelihood ratio test, **** Fisher’s exact test, ^#^ income (in number of basic salaries—basic salary = BRL ± 220—(Brazilian Real)).

**Table 2:** Characteristics of patients colonized with MRSA.

Characteristics	Patient 1	Patient 2	Patient 3
Age (years)	44	33	19
Sex	Male	Female	Male
Duration of dialysis (months)	>240	>120	30
Vascular access	* AVF	* AVF	Permcath
Comorbidities	^#^ CAKUT/Hepatitis C	Red cell aplasia	^+^ SLE
Antibiotic use (previous 12 months)	No	No	Yes
Smoking	No	No	No
Infection (previous 12 months)	No	No	Yes
Hospitalization (previous 12 months)	No	No	No
Corticosteroid use (previous 3 months)	Yes	No	Yes
MRSA resistance pattern	OXA/PN	OXA/PN	^&^ OXA/PN/ERITRO

* Arteriovenous fistula; ^#^ congenital anomalies of kidneys and urinary tract; ^+^ systemic lupus erythematosus; ^&^ oxacillin, penicillin, and erythromycin.

**Table 3:** Antimicrobial resistance and susceptibility of *S. aureus* strains isolated from hemodialysis patients.

Antimicrobials	Number of Patients Colonized by *S. aureus* Strains Out of Total Hemodialysis Population (n = 96)		
	Intermediate	Susceptible	Resistant
Penicillin	0	0	34 (100%)
Oxacillin	0	31 (91.17%)	3 (8.83%)
Linezolid	0	34 (100%)	0
Levofloxacin	0	34 (100%)	0
Gentamicin	0	34 (100%)	0
Clindamycin	0	15 (44.2%)	19 (55.8%)
Erythromycin	0	12 (35.3%)	22 (64.7%)
Teicoplanin	0	34 (100%)	0
Tigecycline	0	34 (100%)	0
Vancomycin	0	34 (100%)	0
Rifampicin	0	34 (100%)	0
* TMP-SMX	0	33 (97.1%)	1 (2.9%)

This table summarizes the resistance, susceptibility, and intermediate responses of *S. aureus* strains isolated from nasal carriers (n = 34) within the hemodialysis population (n = 96). Results are presented as the number of patients colonized by *S. aureus* strains and their respective percentages. * TMP-SMX = trimethoprim–sulfamethoxazole.

## Data Availability

The original contributions presented in this study are included in this article. Further inquiries can be directed to the corresponding author.
